# Management of refractory rectal variceal bleed using computed tomography

**DOI:** 10.6026/973206300200812

**Published:** 2024-07-31

**Authors:** Devara Anil Kashi Vishnuvardhan, Lavanya P, Sandeep Botcha, Biswa Basu Das, Sandeep Sahu, Payala Vijayalakshmi

**Affiliations:** 1 MVP Medicover Hospitals, Visakhapatnam, Andhra Pradesh, India; 2 Department of Microbiology, GITAM Institute of Medical sciences and Research, GITAM Deemed to be University, Visakhapatnam, India

**Keywords:** Refractory rectal variceal bleed, CT guided percutaneous glue injection, CT abdominal angiogram

## Abstract

The management of refractory rectal variceal bleed using a minimally invasive percutaneous approach is described. Rectal varices are portosystemic collaterals that arise as a complication of portal hypertension. Bleeding is less common from rectal varices than from esophageal varices, but it is potentially life-threatening. Hence, it is of interest to describe a novel minimally invasive percutaneous technique to control refractory bleeding from rectal varices in a complex scenario where other proven treatments have failed. In the present study, a 28-year-old male presented to the Emergency department with one episode of hematemesis, hematochezia and severe abdominal pain. Sigmoidoscopy revealed actively bleeding rectal varices. CT abdominal angiogram revealed variceal formation in the rectum. we successfully performed CT guided percutaneous N- butyl cyanoacrylate (NBCA) glue injection of rectal varices with immediate and complete cessation of rectal bleed after failed endoscopic sclerotherapy.

## Background:

Rectal varices are porto-systemic collaterals that arise as a complication of portal hypertension. Despite their significant prevalence among cirrhotic patients, clinically important bleeding occurs only in a minority. Acute rectal variceal bleeding can be massive and life-threatening even though it is rare [1]. The diagnosis and management of bleeding rectal varices remains challenging, and their anatomical diversity makes development of standardized guidelines extremely difficult [2]. Various treatment options are available with endoscopic therapies being widely used initially, while both interventional radiology and surgery are being considered for refractory bleeding rectal varices [3]. Therefore, it is of interest to describe a novel minimally invasive percutaneous technique to control refractory bleeding from rectal varices in a complex scenario where other proven treatments have failed.

## Methodology:

A 28-year-old man presented to the Emergency Department with one episode of hematemesis, hematochezia and severe abdominal pain. He was a diagnosed case of EHPVO, portal hypertension with portal cavernoma, esophageal varices, rectal varices, and underwent splenectomy. There was history of syncopal attacks, vomiting and malena. No history of hypertension or diabetes mellitus. On admission, his vital signs were recorded as heart rate of 115Bpm, BP of 90/60mmHg, SpO2 97%, per abdomen was soft and non- tender, digital rectal examination showed fresh blood. He had severe anemia (hemoglobin 5.6 g/dL) and thrombocytopenia (platelets 1.39 lakh/cubic mm), total bilirubin level of 2.0 mg/dl with deranged liver function tests and coagulation profile. UGIE revealed two small esophageal varices without evidence of active bleeding from upper Gl tract. Sigmoidoscopy revealed actively bleeding rectal varices. Endoscopic sclerotherapy was done with 3ml of setrol injected into the varix showing nipple sign, which achieved temporary hemostasis.

## Results:

**Figure 1:**
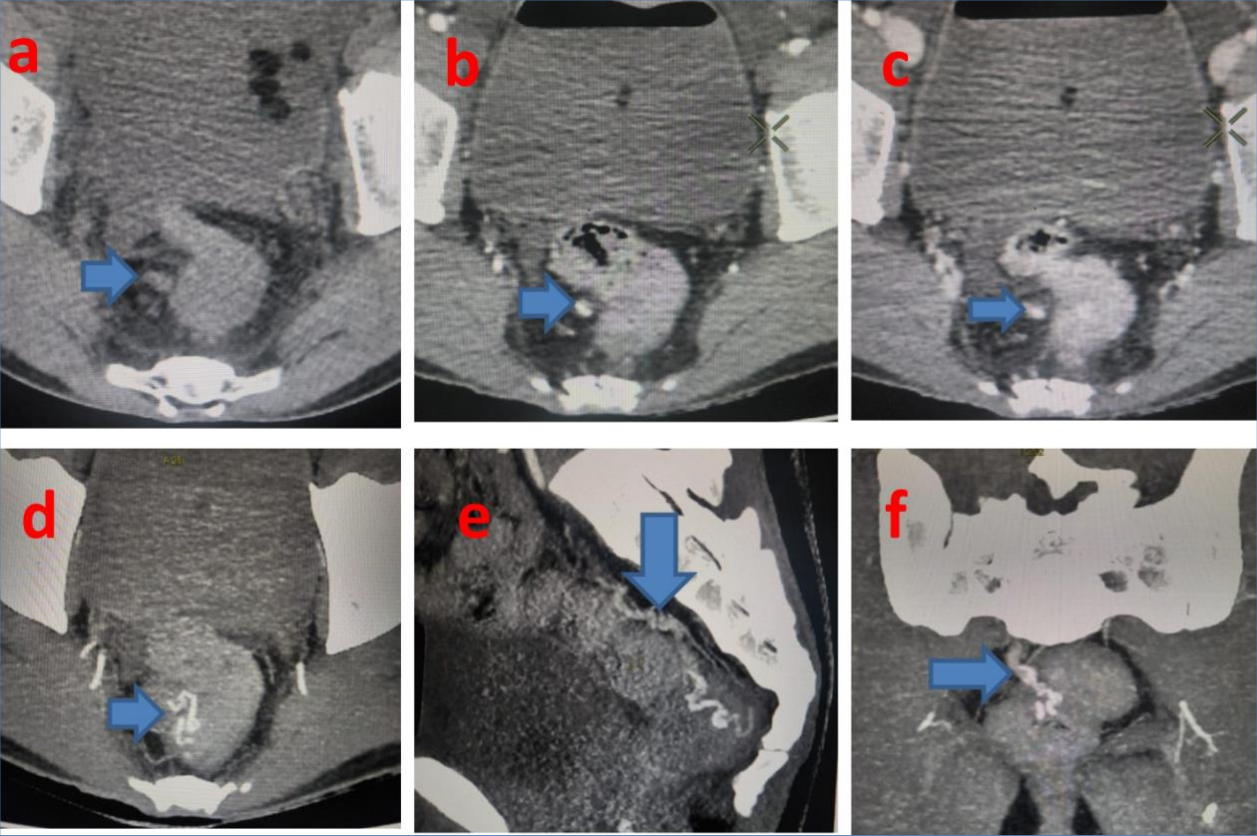
Right pararectal collaterals filling the rectal varix. CT Abdominal angiogram (a-f) revealed variceal formation in the anorectum with features of EHPVO seen as diffuse narrowing of main portal vein and right branch due to chronic portal vein thrombus with portal cavernoma and varices at peripancreatic, mesenteric and anorectal regions.

**Figure 2:**
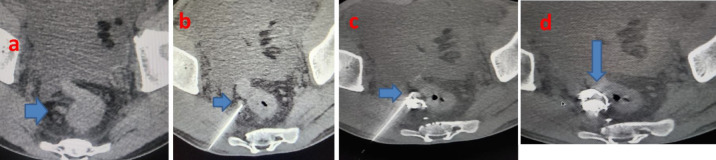
CT Guided percutaneous glue injection of rectal varix: axial nect pelvis. The glue was injected using a 22G spinal needle into the right pararectal and rectal varices (a-d) at three levels under CT guidance with consequent obliteration of rectal varices leading to immediate and complete cessation of bleeding per rectum.

**Figure 3:**
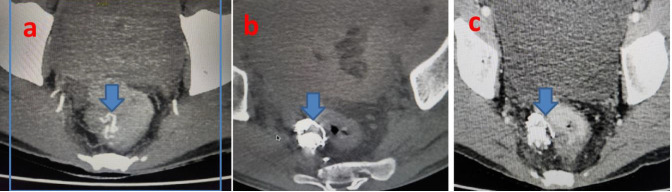
CT Guided percutaneous glue injection of rectal varix: pre-procedure axial CT angiogram of pelvis rectal varix. The CT abdominal imaging (a-c) after the procedure revealed non-opacification of rectal varices.

CT Abdominal angiogram revealed variceal formation in the anorectum (Figure 1a-f) with features of EHPVO seen as diffuse narrowing of main portal vein and right branch due to chronic portal vein thrombus with portal cavernoma and varices at peripancreatic, mesenteric and anorectal regions. Post splenectomy status and incidental note of fusi-saccular aneurysm of size 6mm and saccular aneurysm of size 5mm along the course of proximal and distal segments of splenic artery respectively. Patient was initially managed with nor-adrenaline infusions, blood transfusions along with supportive symptomatic management to correct coagulation profile and liver function tests. Endoscopic injection sclerotherapy (EIS) was attempted without success as patient bled again the next day. In view of inaccessibility of main portal vein due to chronic portal vein thrombus and portal cavernoma, inaccessibility of splenic vein due to post splenectomy status, TIPS, Endovascular Trans-hepatic and Trans-splenic embolization approaches could not be attempted. Hence, based on cross sectional imaging, we performed CT Guided Percutaneous glue injection of rectal varices using 3 ml of 50% cyanoacrylate glue (N-butyl-cyanoacrylate mixed with lipiodal in equal concentrations) under local anesthesia. The glue was injected using a 22G spinal needle into the right pararectal and rectal varices (Figure 2a-d) at three levels under CT guidance with consequent obliteration of rectal varices leading to immediate and complete cessation of bleeding per rectum. Patient tolerated the procedure well. Endoscopy performed after 1 week confirmed a marked shrinkage of rectal varices. CT abdominal angiogram performed 3 months (Figure 3a-c)after the procedure revealed non-opacification of rectal varices. On 12 month follow up there was no recurrence of bleeding per rectum.

## Discussion:

Anorectal varices represent porto-systemic collateral vessels that constitute a pathway for portal venous blood flow between the superior rectal veins of the inferior mesenteric system and the middle, inferior rectal veins of the iliac system [3]. They manifest as dilated and engorged submucosal veins in the rectum. Anorectal varices most commonly result from portal hypertension secondary to cirrhosis. A variety of conditions that result in non-cirrhotic portal hypertension are also associated with the development of anorectal varices, including mesenteric or splenic vein obstruction from carcinoid syndrome or pancreatitis respectively, along with cavernous malformation of the portal vein [4]. Although rare, bleeding from rectal varices can be life threatening. The management of patients with rectal variceal bleeding is not well established. It is important to ensure hemodynamic stability with blood transfusion and to correct any coagulopathy prior to treating the bleeding varices [5]. A variety of treatment modalities have successfully been employed to treat bleeding anorectal varices, including endoscopic therapies like endoscopic injection sclerotherapy (EIS), band ligation (EBL) or obturation (EVO), Interventional radiological procedures (Transjugular intrahepatic portosystemic shunt TIPS, endovascular transhepatic and transpslenic embolization of rectal varices), Surgical procedures (including simple suture ligation or stapled anopexy, mesenteric vein occlusion or porto-caval shunt surgery) [6]. In the current case scenario, with failed endoscopic sclerotherapy, TIPS and percutaneous transhepatic embolization were not possible due to the presence of portal vein thrombosis, portal cavernoma and complex bunch of anorectal variceal formation. Transplenic embolization was not possible owing to splenectomy. After reviewing the CT abdominal angiogram, the right pararectal/anorectal varices were seen as bunch of intertwined collaterals coursing down from the plexus of veins in the pancreatic bed and non-visualisation of inferior mesenteric vein, we planned and successfully performed glue obliteration of the rectal varices by direct puncture access with 22G spinal needle into the right pararectal varices, via the right transgluteal approach under CT guidance. Similar case was described before where the procedure was performed using a hybrid interventional radiography/computed tomography (IVR-CT) system. In this case the right superior rectal vein was punctured with an 18-gauge needle under CT fluoroscopic guidance. Subsequently, a 0.035-inch guidewire (Radiofocus, Terumo, Tokyo, Japan) was inserted toward the feeding route of the varices and a 5F sheath introducer (Super Sheath, Medikit, Tokyo, Japan) was placed at the right superior rectal vein under fluoroscopic guidance followed by embolization of bilateral superior rectal veins using a steerable catheter. Comparatively, in our case there were right pararectal bunch of intertwined collaterals which couldn't be cannulated owing to small size. Hence, introduction of sheath, micro catheter, DSA was not possible during embolization and entire procedure was performed successfully under CT guidance only. Limitations like failure to inject glue into the anorectal varix, inadvertent injury to the rectal wall, pelvic hematoma; pelvic abcess formation should be considered while performing CT guided percutaneous glue injection of the rectal varices.

## Conclusion:

Navigating the intricate anatomy of extra-hepatic portal venous obstruction helps in managing severe rectal variceal bleeding. We show that optimal utilization of CT guided percutaneous glue injection technique can achieve timely resolution and should be considered in the management of such patients with refractory rectal variceal bleed.

## Sources of support:

Nil
